# Decoding the complexity of metastasis

**DOI:** 10.20892/j.issn.2095-3941.2022.0031

**Published:** 2022-03-14

**Authors:** Yingcheng Wu, Tiancheng Zhang, Xiaoming Zhang, Qiang Gao

**Affiliations:** 1 Department of Liver Surgery and Transplantation, Key Laboratory of Carcinogenesis and Cancer Invasion (Ministry of Education), Liver Cancer Institute, Zhongshan Hospital, Fudan University, Shanghai 200032, China; 2 The Center for Microbes, Development and Health, Key Laboratory of Molecular Virology and Immunology, Institute Pasteur of Shanghai, Chinese Academy of Sciences, Shanghai 200031, China; 3 Key Laboratory of Medical Epigenetics and Metabolism, Institutes of Biomedical Sciences, Fudan University, Shanghai 200032, China; 4 State Key Laboratory of Genetic Engineering, Fudan University, Shanghai 200433, China

Cancer metastasis remains one of the most confounding questions in oncology^1,2^. Although current cutting-edge techniques enable very early detection of tumors, profiling whether a tumor has already begun to spread and where it has attempted to colonize remains a major hurdle. Indeed, metastatic seeding events exhibit remarkable temporal and spatial heterogeneity, wherein the origin (primary site) and destination (metastatic site) are highly dynamic. For example, liver metastasis is particularly common and remains a leading cause of mortality^3^. Primary cancers are diverse and can include gastrointestinal cancer, such as colorectal cancer and pancreatic cancer, as well as cancers of extraperitoneal origin, such as breast cancer, which has heterogeneous clinical phenotypes and a range of therapeutic responses. Because existing knowledge of cross-tissue metastasis remains far from complete, the generation of a unified pan-cancer metastasis map remains a pressing need. In this perspective, we outline the growing advances in metastasis biology, with a focus on the challenges in using high-throughput technologies and the state-of-the-art theories among the metastasis research community.

## The nature of metastasis

Metastasis has long been appreciated to be the most lethal complication of cancer progression^4^. This process requires several key steps involving cancer cells leaving the tumor, entering the vessels, surviving the systemic immune system, and finally colonizing distal organs^5^. These key aspects of cancer seeding are regarded as the hallmarks of metastasis^6^. In detail, at the initial stage of metastasis, cancer cells often have high motility and invasiveness, thus enabling later intravascular dissemination and transport^7^. Interestingly, the balance between circulating cancer cells and the systemic immune system can determine the fate of metastatic cells^8^. Later, at the colonization stage, cancer cells show complex interactions with the microenvironment^5,6^. For example, hepatic metastatic cells engage in cross-talk with a broad spectrum of liver resident cells (e.g., hepatic stellate cells, Kupffer cells, and inflammatory cells), which have opposing roles in the progression of metastasis^9^. Recent evidence has also highlighted that the primary tumor provides a microenvironment that can precondition metastatic cancer cells, thus favoring their growth and disrupting the fragile balance^10^. The most recent high-throughput omics technologies can now be leveraged to profile metastatic cell landscapes across different conditions and cancer types, thus yielding data-driven insights to refine understanding of the nature of metastasis.

## Not all metastatic cancer cells are equal

In the context of metastasis biology, an important question naturally arises: how do metastatic cancer cells evolve after arriving at a new location? Paradoxically, genomic profiling has indicated that primary and metastatic cells generally have similar profiles^11–16^. For instance, hepatic metastatic cancer cells inherit multiple genetic subclones from primary colorectal cancer cells^11^. Moreover, ~65% of metastases have been reported to originate from independent subclones, thus highlighting the complicated seeding patterns of colorectal cancer cells^17^. The route of colorectal hepatic metastasis has been classified into 3 models: initiation from primary tumors (~62.3%), lymph node metastases (~36.1%), and intrahepatic metastases (~1.6%)^18^. However, almost all these findings were based on a “gene-centric” model, which does not consider the effects of epigenetic cis-regulatory regions. Further exploration of whether the alterations occur at enhancers or transcription factor binding sites should provide a unique integrated perspective on the complicated linkage between the epigenome and aggressive phenotypes.

Beyond genetic diversity, another confounding question regarding metastasis is the evolution of cancer cell state. Colonized cancer cells are likely to be “unfamiliar” with the new environment and may potentially initiate an adaptation program. For example, in the liver, the central organ of glycolytic and drug metabolism, metastatic cancer cells are exposed to toxins and nutrients. Consequently, these metastatic tumors exhibit strong activation of drug metabolism and PPAR signaling pathways^16^. At the epigenetic level, hepatic metastatic cancer cells shift their gene transcription programs and show reprogramming of the landscape of enhancer binding by liver-specific transcription factors^19^. Another independent group has also reported that metastatic cells undergo chromatin remodeling in the liver^20^, thereby highlighting the specific transcriptional states shaped by the liver microenvironment.

These recently generated data have raised more hypotheses than they have answered. Many microenvironmental factors (i.e., metabolites and stromal cells) appear to associate with cancer cell phenotypes, but little is known regarding whether those changes are passengers or drivers. The overall transcriptional state is reprogrammed, but whether this state is dominated by the majority or minority of cancer cell subpopulations remains an interesting question to be answered. The resolution of these fundamental questions should open exciting new paths to in-depth understanding of metastatic cell states and regulation, an area of remarkable importance in both metastasis biology and anti-metastasis therapy.

## Charting the evolution of the metastatic microenvironment across space and time

Along with (epi)genomic, transcriptomic, and proteomic dysregulation, microenvironmental alterations have emerged as a hallmark of metastasis^6,7^. Mouse model evidence has indicated that liver metastasis creates an “immune desert” environment with significantly diminished T cell diversity and function^21^. That work has explained why immunotherapy appears to fail when metastasis occurs and has raised the possibility of combining radiotherapy with immunotherapy to promote antitumor immunity^21^. We and Li’s laboratories have recently reported the spatiotemporal immune landscape of colorectal cancer liver metastasis^22,23^. We observed that the hepatic metastatic microenvironment is enriched in MRC1^+^ CCL18^+^ M2-like macrophages. Unexpectedly, these macrophage subsets show a broad range of activated metabolism related pathways. To address the challenges of investigating single-cell metabolism, we have developed the pipeline *scMetabolism* (R package: https://github.com/wu-yc/scMetabolism; online version: http://cancerdiversity.asia/scMetabolism/) and used it to confirm the sharp increase in metabolic activity, such as phenylalanine metabolism. These patient-derived observations are consistent with our mouse model data^22^, thus indicating that metastasis-specific immune cell subsets and immunometabolism might potentially be conserved across species.

A crucial step in cancer seeding is the establishment of a pre-metastatic niche (PMN) whose formation can be divided into 4 distinct steps: priming, licensing, initiation, and progression^24^. In the context of liver metastasis, several immune components have been demonstrated to be essential for the PMN, such as neutrophils^25^ and macrophages^26^. However, almost all evidence has originated from laboratory conditions. Higher-level evidence from clinical samples remains needed, although acquiring such samples and defining the PMN state remains challenging. Innovations such as high-resolution imaging (detection of PMN structural changes) and liquid biopsy (detection of circulating biomarkers predictive of early metastasis) are expected to markedly improve the possibility of profiling the PMN and thus deepen understanding of their functional outcomes.

## Opening the black box of how metastasis responds to therapy

Although therapeutic strategies against metastasis are highly diverse across tumors, the consensus is that most metastases significantly decrease the efficacy of existing treatments^27,28^. The importance of combinational therapy (e.g., for advanced breast cancer^29^) or neoadjuvant therapy (for downstaging advanced colorectal cancer^3^) against metastases is increasingly being recognized. In contrast, independent studies have shown that neoadjuvant therapy may also initiate the metastasis cascade by driving the formation of an unfavorable metastatic microenvironment^30^ and inducing the generation of resistant cancer stem cells^31^. These seemingly paradoxical observations highlight the complexity of metastatic cancer cell fitness with respect to their environmental conditions and prompt the fundamental question of how to trace metastatic seeding under distinct pharmacological conditions.

To overcome this challenge, our group and Li’s group have recently used scRNA-seq to compare the immune microenvironment of neoadjuvant chemotherapy-treated and treatment-naïve colorectal cancer liver metastasis samples^22,23^. Our results have shown that neoadjuvant chemotherapy restores the balance of anti-tumor immunity by depleting MRC1^+^ CCL18^+^ macrophages and increasing cytotoxic CD8^+^ T cells in responsive patients. In contrast, progressive disease and stable disease samples show higher proportion of suppressive immune cells. This result is generally consistent with Li’s findings^23^. These data collectively emphasize the importance of infiltrated immune cells to the sensitivity of neoadjuvant chemotherapy and suggest potential opportunities to explore the mechanisms of metastasis-immune interaction *via* single-cell profiling and computational modeling. Therefore, to improve knowledge of why chemotherapy selectively benefits certain subsets of patients, understanding of not only the cancer cells themselves but also their interaction dynamics across time and space is necessary.

However, knowledge regarding the mechanisms underlying metastatic drug responsive processes remains scarce. Although scRNA-seq can capture the transcriptomes and cell states of both immune cells and cancer cells, an essential question remains: do the immune cells and cancer cells co-evolve during therapy? Moreover, how does neoadjuvant chemotherapy reprogram the state of cancer cells? The local ecosystem of metastatic tumors can vary dramatically during long-term exposure to therapeutic stimulation, wherein alterations in the blood supply, cancer cell and immune metabolism, and intercellular cross-talk are all likely to reshape the therapeutic responses. Thus, longitudinal follow up and profiling of the metastatic ecosystem will be necessary in the future.

## Where we should go from here?

**Figure 1:**
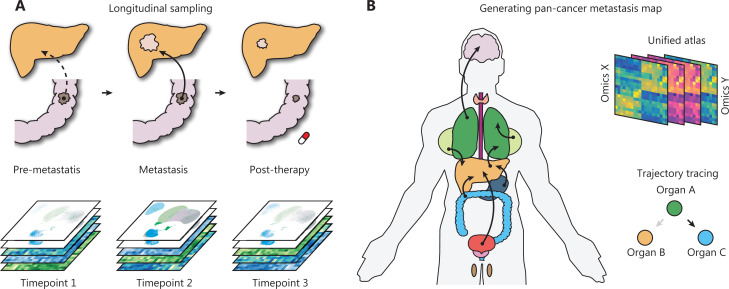
Advances toward a systematic understanding of metastasis. (A) The longitudinal sampling strategy will be informative for the understanding of synchronous and metachronous cancer metastasis. (B) Generation of a large-scale pan-cancer metastasis atlas will aid in drafting a metastasis atlas in the years to come.

First, the longitudinal collection of clinical metastatic samples and their matched primary tumors is particularly informative for identifying the evolutionary patterns of metastasis (**Figure 1A**). Although several efforts have attempted to decode the genetic evolution during metastasis across common cancer types^13,32^, the major goal of decoding the route of metastasis remains far from being met. For example, few studies have focused on explaining why metastases in the same organ can originate from distinct primary cancers. Similarly, why the same primary tumors can colonize different distal organs and how those metastases are specifically transcriptionally reprogrammed are poorly understood. The necessary pan-cancer sampling strategy is practically challenging, because it requires long-term collection of non-invasive biopsy samples; however, it may make finally assembling an atlas possible.

Second, current computational and statistical tools remain insufficient to model the seeding routes. The complexity of multi-omics data makes them powerful but also difficult to interpret. The metastasis research community is producing increasingly larger datasets, covering multiple dimensions (e.g., spatial omics) across multiple timescales (e.g., longitudinal sampling). Future technological advances are likely to improve the throughput, dimension, and resolution. Therefore, bioinformatics algorithms must urgently be updated to analyze such large data. We believe that future *in silico* construction of metastatic trajectories will answer essential questions, such as whether metastatic evolution is discrete or continuous.

Third, a more advanced lineage tracing tool remains needed to track metastatic seeding. The current Cas9-based single-cell tracing system enables monitoring of metastatic cells with months of growth and dissemination^33,34^. However, those methods largely rely on the construction of gene-edited cell line models. With patient-derived xenografts and organoids, which can closely reflect the heterogeneity of clinical samples, the genotypes and phenotypes of human metastatic cancer cells can now be charted. We believe that the surge in of advanced high-throughput sequencing technologies and lineage tracing systems will advance metastasis biology beyond the current paradigms.

Finally, the development of a pan-cancer metastasis resource will support a broad spectrum of metastasis research and enhance the rapid discovery of novel metastasis biology (**Figure 1B**). The future database or resource may potentially enable the following: (1) systemic trajectory tracing of primary and metastatic cancers; (2) unified analysis of multi-omics primary-metastasis data; and (3) drug response prediction based on the multi-omics profile. We believe that these efforts will be particularly valuable for the analysis of multi-omics big data and new hypothesis generation.

Metastasis research is rapidly advancing. The surging advances in single-cell spatial omics, computational biology, and data science are providing unprecedented opportunities for data generation, analysis, and theoretical development. Progress in exploring the molecular principles controlling cancer seeding will advance the field from viewing a fixed snapshot to a dynamic perspective on metastatic evolution. Simultaneously, the generation of unbiased, large-scale, high-throughput data is poised to facilitate the generation of a map of pan-cancer metastasis and is expected to drive an exciting research paradigm shift in the coming years.
